# Generation of Viable Plant-Vertebrate Chimeras

**DOI:** 10.1371/journal.pone.0130295

**Published:** 2015-06-30

**Authors:** Marjorie Alvarez, Nicole Reynaert, Myra N. Chávez, Geraldine Aedo, Francisco Araya, Ursula Hopfner, Juan Fernández, Miguel L. Allende, José T. Egaña

**Affiliations:** 1 FONDAP Center for Genome Regulation, Facultad de Ciencias, Universidad de Chile, Santiago, Chile; 2 Dept. of Plastic and Hand Surgery, University Hospital rechts der Isar, Faculty of Medicine, Technische Universität München, Munich, Germany; 3 Laboratory of Developmental Cell Biology, Department of Biology, Facultad de Ciencias, Universidad de Chile, Santiago, Chile; 4 Institute for Medical and Biological Engineering, Schools of Engineering, Medicine and Biological Sciences, Pontificia Universidad Católica de Chile, Santiago, Chile; Santa Fe Institute, SPAIN

## Abstract

The extreme dependence on external oxygen supply observed in animals causes major clinical problems and several diseases are related to low oxygen tension in tissues. The vast majority of the animals do not produce oxygen but a few exceptions have shown that photosynthetic capacity is physiologically compatible with animal life. Such symbiotic photosynthetic relationships are restricted to a few aquatic invertebrates. In this work we aimed to explore if we could create a chimerical organism by incorporating photosynthetic eukaryotic cells into a vertebrate animal model. Here, the microalgae *Chlamydomonas reinhardtii* was injected into zebrafish eggs and the interaction and viability of both organisms were studied. Results show that microalgae were distributed into different tissues, forming a fish-alga chimera organism for a prolonged period of time. In addition, microscopic observation of injected algae, *in vivo* expression of their mRNA and re-growth of the algae *ex vivo* suggests that they survived to the developmental process, living for several days after injection. Moreover microalgae did not trigger a significant inflammatory response in the fish. This work provides additional evidence to support the possibility that photosynthetic vertebrates can be engineered.

## Introduction

In 1772 Joseph Priestley demonstrated the dependency of animal survival on photosynthesis. He showed that a mouse died if it was placed in a closed compartment but it survived if a plant was introduced as well. Priestley concluded that plants restore whatever breathing animals removed from the environment [1]. Later, it was established that oxygen was the molecule released by plants that was required by animals. This strong dependency of animals on a continuous external supply of oxygen is in contrast to other key molecules for cell metabolism that can be stored in specialized tissues. For instance, calcium is stored in bone and energy in fat tissue, thus conferring a degree of autonomy to animals as to their acquisition of an external supply of these metabolites. This issue leads to the evolutionary question of why animals do not produce oxygen by themselves. It is possible to speculate that exposure of animals to sunlight also exposes them to predators, high temperatures, and other dangers thus representing a selective drawback. Moreover, in order to allow light penetration, animals would need to be transparent; however in most animal species, the integument includes pigments, hair or feathers. Additionally, such free oxygen may cause toxic damage by the formation of reactive oxygen species and finally, after oxygen became abundant in the atmosphere and oceans, animals have thrived using the unlimited supply of this gas and have developed various ways to absorb it and distribute it to tissues. Of course, with increasing size and tissue complexity, this requirement has become more difficult to fulfill.

Although the vast majority of animals do not produce significant amounts of oxygen, a few exceptions have proven that photosynthetic capacity is physiologically compatible with animal life. Such rare phenomena have caught the attention of scientists since long ago, and reports as early as the 19^th^ century described the presence of green pigments in animals [2]. Special mechanisms to establish symbiotic relationships with unicellular algae or cyanobacteria have appeared in animals of a few orders (Mollusca, Porifera, Cnidiaria, Acoelomorpha and Chordata;[3]). Probably the best studied photosynthetic animal is the sea slug *Elysia chlorotica*, which evolved a camouflage strategy based on the incorporation of chloroplasts, which are phagocytically introduced into specific intestinal cells. As consequence, *E*. *chlorotica* not only looks like a plant but also is photosynthetically active, fixing carbon and releasing oxygen in the presence of light [4]. Interestingly, *E*. *chlorotica* can survive for several months in captivity without an external food supply, as long as it is exposed to light. In any case, such photosynthetic animals seem to be restricted to a few non-vertebrate aquatic organisms. Here, we ask whether it could be possible to incorporate photosynthetic eukaryotic cells into a vertebrate animal model. In the present work, the microalga *Chlamydomonas reinhardtii* (*C*. *reinhardtii*) was injected into zebrafish eggs and the interaction and viability of both organisms was studied.

## Materials and Methods

### Zebrafish breeding

Zebrafish embryos (*Danio rerio*) from the wild type AB strain or the transgenic strain *BACmpo*::*GFP* [5] were obtained from our breeding colony. All embryos were collected by natural spawning and raised at 28.5°C in E3 medium (5 mM NaCl, 0.17 mM KCl, 0.33 mM CaCl_2_, 0.3 mM MgSO_4_, and 0.1% methylene blue, adjusted to pH 7.0) in Petri dishes[6]; E3 medium was changed as needed. Embryonic and larval ages are expressed in hours or days post-fertilization (hpf or dpf). Incubations were carried out for the required time under constant light. All animals used in this work were anesthetized with MS-222 (Tricaine, A5040, Sigma, St. Louis, MO, USA) before each experiment. All procedures were approved were by the Animal Ethics Committee of the Universidad de Chile.

### Cell culture of *Chlamydomonas reinhardtii*

The cell-wall deficient *Chlamydomonas reinhardtii* (*C*. *reinhardtii*) strain UVM4 [7] was grown photomixotrophically in liquid TAPS-medium (Tris Acetate Phosphate, supplemented with 1% (w/v) sorbitol; [8]) under continuous light exposure (30 μE/m-2/s-1) and room temperature. Cell concentration in the culture was determined by a Neubauer chamber.

### Injection of *C*. *reinhardtii* into zebrafish embryos

*C*. *reinhardtii* cells were suspended in TAPS medium at different concentrations (750, 2,500 and 10,000 algae/μl), loaded into glass capillary needles and microinjected (Microinjector MPPI-2 Pressure Injector, Applied Scientific Instrumentation, Eugene, OR) into the yolk sphere of zebrafish embryos at 2 different stages of development: 0 and 24 hpf. Next, fish embryos and larvae were raised under constant light conditions and fish survival was evaluated daily for up to 5 days using a dissecting microscope. Injections with only TAPS medium were used as control and results were expressed as percentage of survival.

### Distribution of *C*. *reinhardtii* into zebrafish embryos and larvae

After injection (0 hpf), *C*. *reinhardtii* cells were visualized either by their green color or the red auto-fluorescence of chlorophyll. For imaging, living zebrafish embryos and larvae were examined using an epifluorescence-inverted microscope (Olympus scanR, Olympus Biosystems, Munich, Germany), equipped with a motorized stage. Embryos were randomly chosen, mounted and anesthetized in 0.75% low-melting point agarose containing 5% Tricaine (Sigma, St. Louis, MO) in a 35 mm imaging dish and placed in a lateral position. Z-stack images were taken at a fixed 10 μm intervals in a confocal microscope (Zeiss LSM 510 Meta, Carl Zeiss AG, Oberkochen, Germany). Z-Projections of the stacks were then merged and combined to generate a mosaic image of the whole fish using the software Zeiss LSM Image Browser Version 3.1.0.99, ImageJ 1.46r (Java 1.6.0_20 (64-bit)) and Adobe Photoshop CS6. For localization of algae, 16 cell stage zebrafish embryos (1.5 hpf) were fixed and stained with an anti-β-catenin antibody (polyclonal, 1:100, Sigma) as described before [9]. For cell tracking, injected embryos were quickly placed under an inverted fluorescent microscope equipped with a Z motor (Prior Scientific Instrumentation, Cambridge, UK) and a chilled CCD camera (Hamamatsu C5985, Japan). Image grabbing and analysis were performed using the Metamorph software (Molecular Devices, Sunnyvale, CA). Time-lapses were taken every 2 minutes and the videos examined with the Metamorph software.

### Viability of the algae *in vivo*

In order to evaluate the metabolic activity of algae in 3 dpf/ dpi fish, larvae were stored at -80°C in RNAlater (Qiagen, Hilden, Germany). After homogeneization with a pestle, total RNA isolation was performed with a highly pure RNA isolation kit (Roche Applied Science GmbH, Mannheim, Germany). To determine the expression of the algae psbD gene, the Kit Transcriptor One Step RT-PCR (Roche Applied Science GmbH, Mannheim, Germany) was used. Primer sequences for psbD were: 5’-GCCGTAGGGTTGAATG-3’ and 5‘-GTTGGTGTCAACTTGGTGG-3’. Fish β-actin was chosen as housekeeping gene: 5’- CCCAGACATCAGGGAGTGAT-3’ and 5’- TCTCTGTTG GCTTTGGGATT -3’.

### Viability of the algae *ex vivo*

In order to evaluate the viability of the algae inside the fish, 0 hpf embryos were injected with algal TAPS-medium or an algae cell-suspension (2500 cells/μl) and raised under the same conditions. 3 dpi larvae were selected and anesthetized with 5% Tricaine (Sigma, St. Louis, MO). Then, embryos were placed in a 100 μm cell-strainer and washed thoroughly with Hank’s solution (Sigma, St. Louis, MO, USA) to remove possible externally adhered algae from their bodies. Embryos were disintegrated in Trypsin-EDTA (1x containing 0.025% trypsin and 0.01% EDTA, Gibco, Thermo Fisher Scientific, MA, USA) for 15 min at 37°C using a 1 ml syringe. The reaction was stopped with L15 medium (Sigma, St. Louis, MO, USA) supplemented with 10% fetal bovine serum (FBS, Gibco, Thermo Fisher Scientific, MA, USA) and the final cell suspension filtered through a Nytal filter (35 μm pore size; Sefar AG, Heiden, Switzerland). Cells were centrifuged (5 min, 300 g) and resuspended in TAPS-medium supplemented with 10 μg/ml Paramomycin (Sigma, St. Louis, MO, USA). Algae were allowed to grow for a minimum of five days in the liquid culture, and then plated over a TAPS-agar plate with the same concentration of antibiotics.

### Innate immune system interaction assay

To monitor the interaction of *C*. *reinhardtii* with the zebrafish innate immune system, embryos derived from the *BACmpo*::*GFP* transgenic fish line [5] were injected at 0 hpf with *C*. *reinhardtii* and the distribution of the alga was followed in the larvae up to 5 days. Observation of *C*. *reinhardtii* cells and innate immune neutrophils was carried out using a confocal microscope as described before in this section.

### Functional effects of *C*. *reinhardtii* in zebrafish embryos and larvae

The effects of *C*. *reinhardtii* on functional parameter of the larvae were followed daily for up to 4 days. The size and shape of the injected embryos were compared to the non-injected control embryos at the same developmental stage. Normal cardiac rhythm was considered to be 125 heart-beats/ min. Less than 10% edema was considered normal and the startle response was determined after mechanical stimulation. Finally, eye movements were considered normal simply when they occurred fast. The startle and eye movement responses were measured only at 3 and 4 dpf. All observations were performed using a stereoscope (MVX10, Olympus).

### Statistical analysis

Statistical comparisons were made by using Kruskal-Wallis nonparametric ANOVA with Dunn’s post-test adjustment. Data were collected from at least five independent experiments and showed as average ± SEM. Results were considered significant when p ≤ 0.01.

## Results

### Injection of microalgae into zebrafish eggs

**Fig 1:**
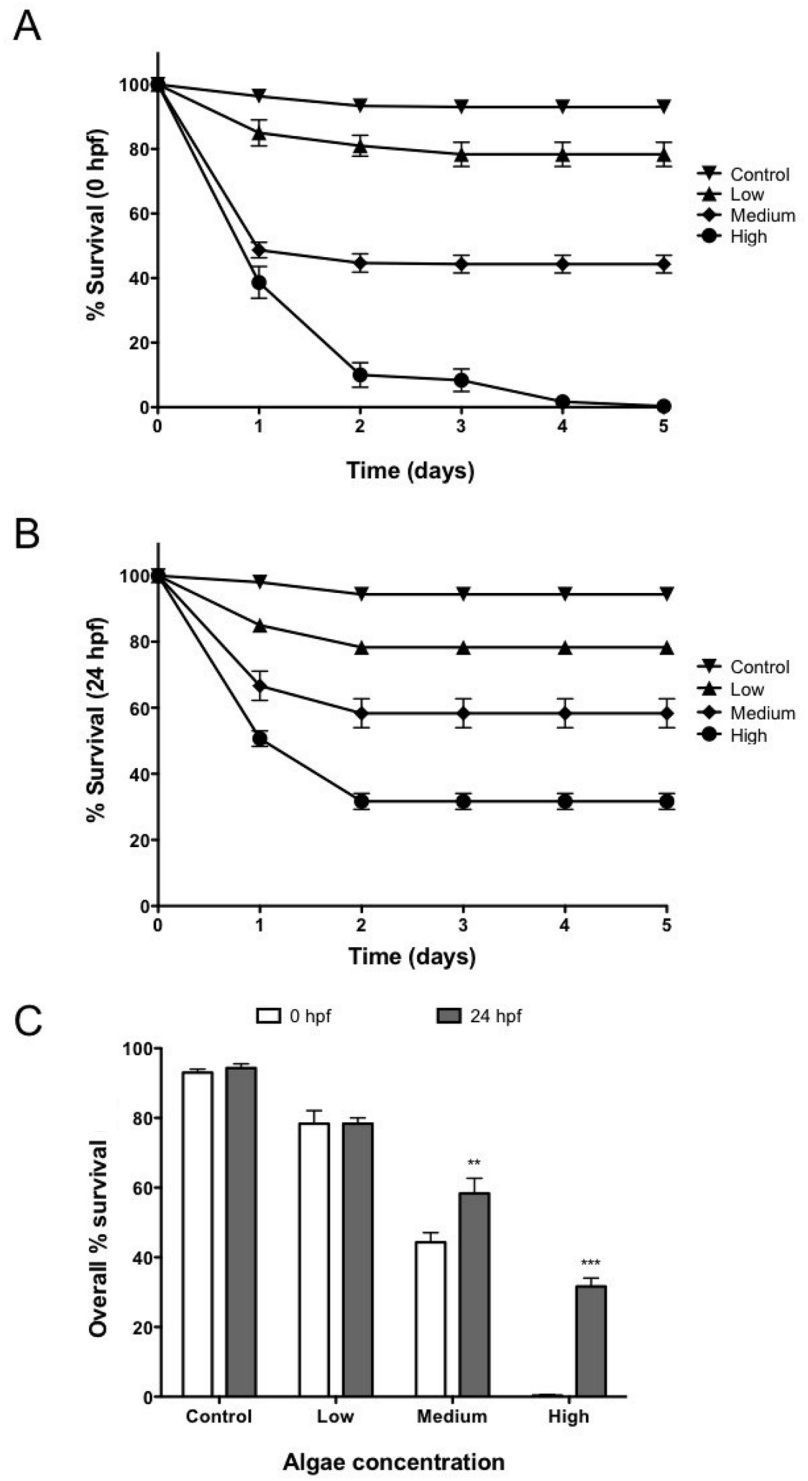
Embryo survival after injection of algae. In order to evaluate the effect of algae in the embryo survival 3 different concentrations of *C*. *reinhardtii* (Low: 750 algae/ μl; medium: 2,500 algae/ μl and high: 10,000 algae/ μl) were injected into zebrafish embryos at 0 hpf (A) and 24 hpf (B). In both embryonic stages, results show a significant decrease in embryo survival with increasing concentrations of algae (C). In most cases a significant mortality was observed only the first days after injection. Error bar represents SEM. ** p ≤ 0.01; *** p≤ 0.001. n = 90 per group.

Our first goal was to optimize the amount of algae injected in early stage zebrafish embryos. For this purpose, suspensions of *C*. *reinhardtii* cells were microinjected into 0 hpf or 24 hpf zebrafish at concentrations of 750, 2,500 and 10,000 algae/ μL. In addition, mock-injected (only algae medium) fish were used as controls. Observation of embryos showed that their mortality was proportional to the concentration of microinjected algae at both stages and occurs only within the first 2 days post injection (Fig 1). At the lowest concentration (750 algae/ μL), no difference in embryos survival was observed between 0 hpf and 24 hpf injected groups. In contrast, when algae were injected in concentrations of 2,500 and 10,000 algae/ μL, a significantly higher survival rate was observed for the 24 hpf group (Fig 1C).

### Embryonic development in the presence of *C*. *reinhardtii*

**Fig 2:**
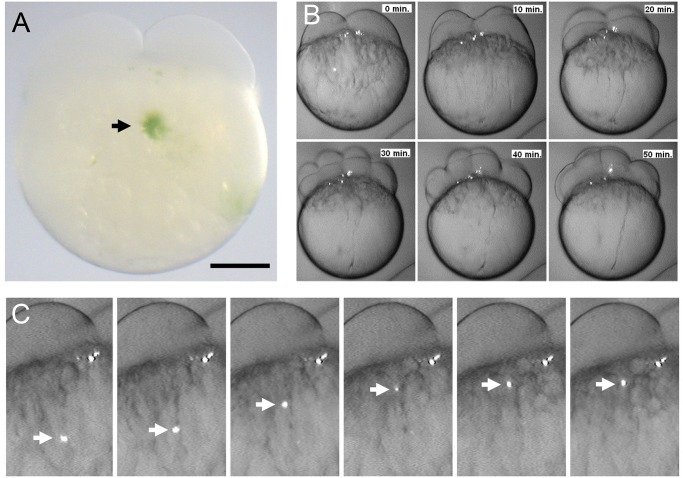
Microinjection of *Chlamydomonas reinhardtii* into the zebrafish yolk. *C*. *reinhardtii* were injected in the middle upper part of early embryos and a green spot was clearly observed at the injection site (A, black arrow). After injection a rapid movement of algae toward the animal pole was observed. Whithin the first 10 minutes most of the algae were quiclky acumulated in the blastodisc and as early as 20 minutes after injection single alga moved to the blastodisc boundary zone (B). The white arrow follows the movement of algae every 30 seconds (C). Scale bar represents 200 μm. n ≥ 100.

**Fig 3:**
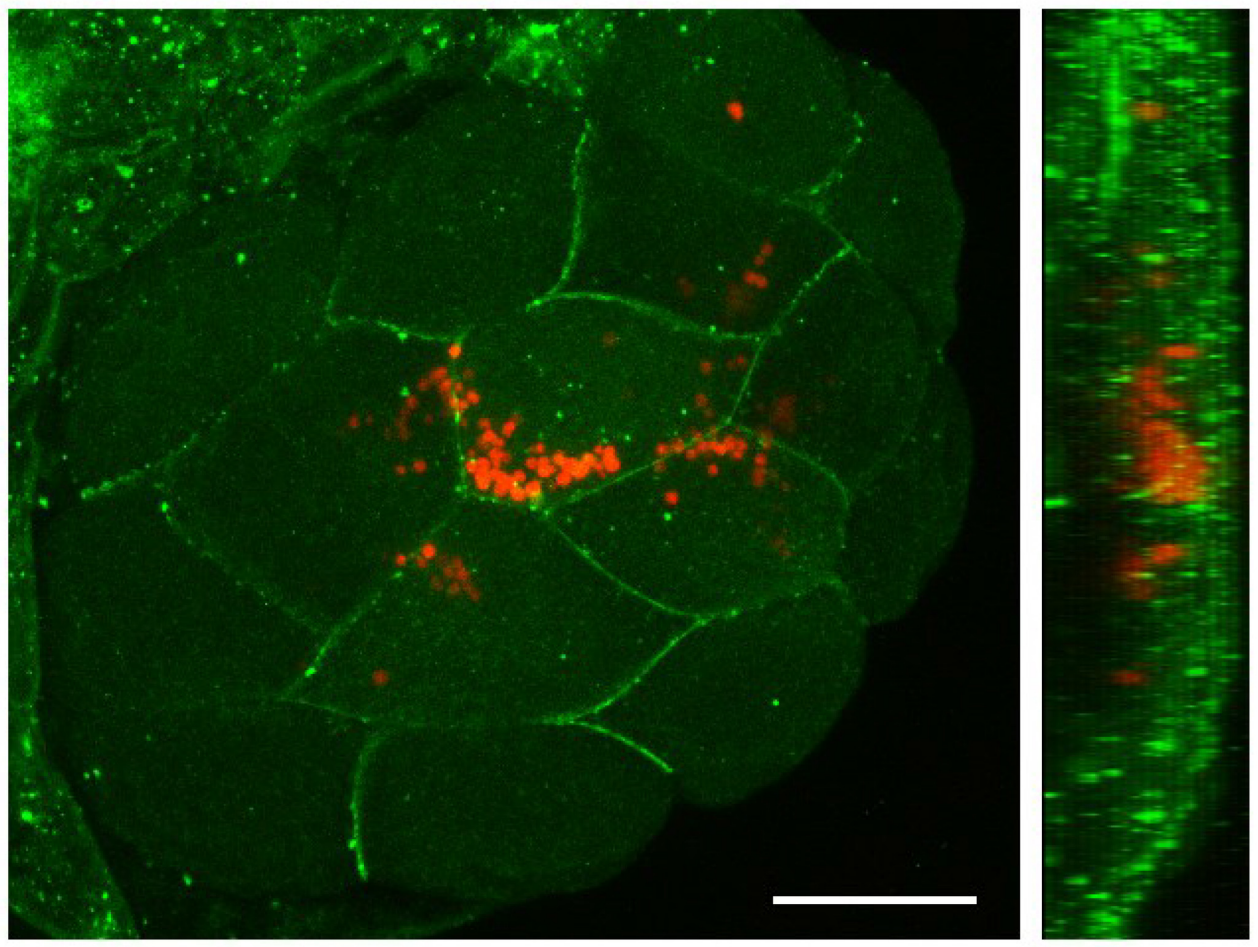
Distribution of *Chlamydomonas reinhardtii* in early zebrafish embryos. A zebrafish embryo at one cell stage was injected with a suspension of algae, raised to the 16 cell stage (1.5 hpf) and processed for immunohistochemistry. The blastoderm was imaged under confocal microscopy to reveal that microalgae were mainly located intracellularly. Cell membranes stained with anti-β-catenin antibody are shown in green, while *C*. *reinhardtii* is observed in red (autofluorescence). A Z-stack projection is shown on the left and a reconstructed Y projection view of the same embryo is observed on the right. Scale bar represents 200 μm. n ≥ 10.

After determining that significant survival of fish can be achieved by injecting suspensions of 2,500 microalgae/μl in 0 hpf embryos, we decided to evaluate the behavior and distribution of *C*. *reinhardtii* during early stages of fish development. We could follow the fate of *C*. *reinhardtii* because of the autofluorescence of cholorophyll, permitting easy imaging of non-transformed algae within the fish embryo and larva. Additionally, due to its green color, injected algae were easily identified by light microscopy (Fig 2A). We detected microalgae in the fish yolk cell immediately after injection at the one cell stage and followed them until the early blastula stage using time-lapse fluorescent microscopy (Fig 2B). A fast movement of algae towards the animal pole of the egg (Fig 2B and 2C and S1 Movie), followed the directional streaming of cytoplasm that precedes the first cleavage [9, 10]. Most *C*. *reinhardtii* cells were carried towards the animal pole while some clusters of microalgae remained within the yolk cell. Microalgae that moved to the blastodisc, became distributed among the blastomeres and remained there during cleavage stages (Fig 2B and 2C and S1 Movie). A Z stack projection of optical confocal sections, showed that algae were mainly surrounded by the membranes of the fish cells (Fig 3A) and were both located at the same confocal plane (Fig 3B), suggesting that at this stage (16 cells; 1.5 hpf) the algae resided intracellularly.

Embryos that had stably incorporated *C*. *reinhardtii* into the blastomeres, yolk cell, or both, continued to develop normally and reached gastrulation. We next evaluated whether the presence of microalgae affected axis formation or morphogenesis in general of the embryo and larva. The size and shape, cardiac rhythm, presence of heart edema, the startle response and eye movements were examined as described in the material and methods section. Results showed that, 1 day after injection, more than 50% of the surviving embryos that contained *C*. *reinhardtii* were normal for the embryonic parameters analyzed. Interestingly at 3 dpi no abnormalities were found in surviving embryos in the following parameters: size and shape, cardiac rhythm, edema, startle response and eye movements.

### Distribution and survival of microalgae in larval stages

**Fig 4:**
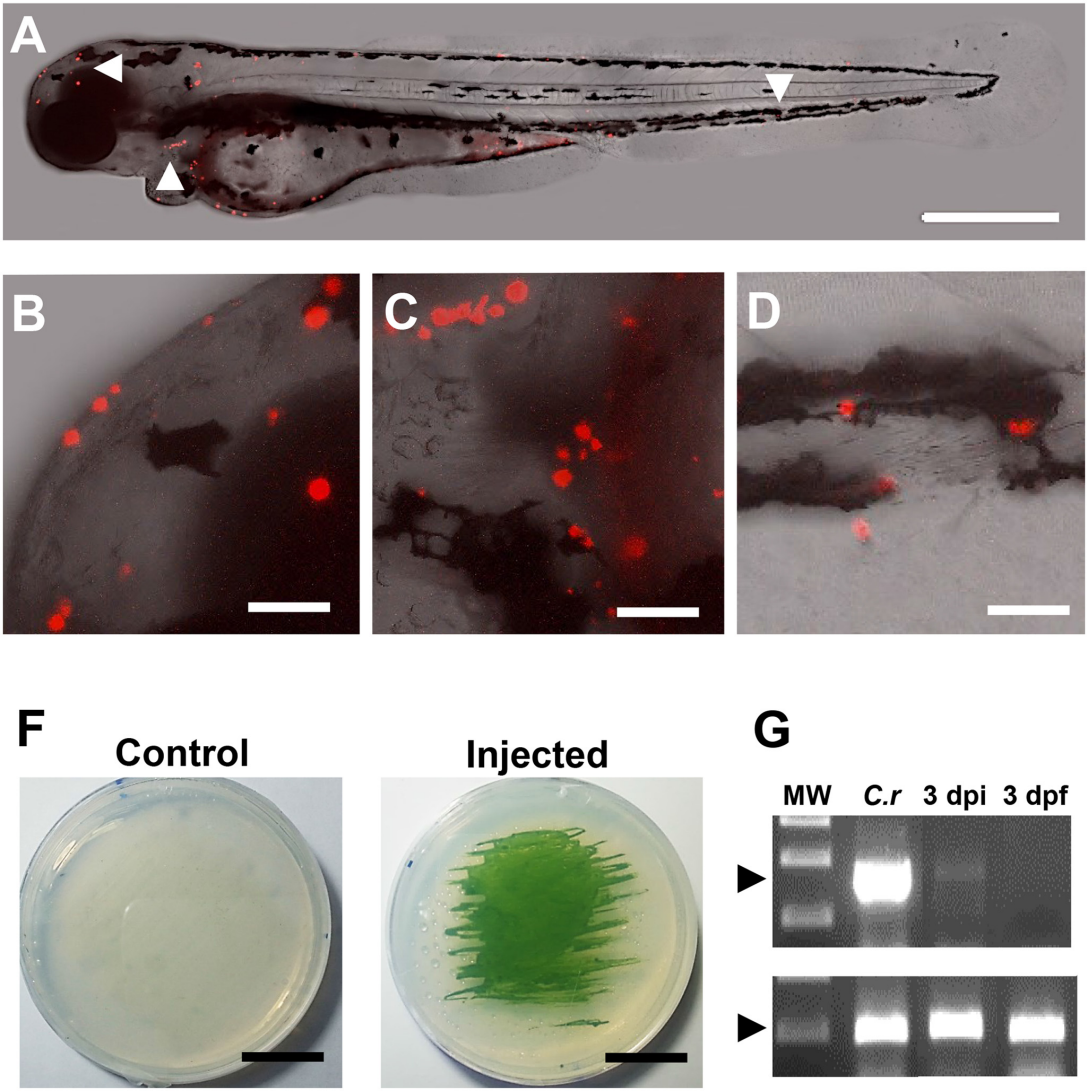
Distribution and viability of microalgae in the zebrafish larvae. *C*. *reinhardtii* was microinjected and visualized at 3 dpf. Results shows that algae distribute along the whole larva (A), including anterior (B), meddle (C) and posterior areas (D). At 3 days post fertilization (3 dpi), injected or control embryos were disaggregated and placed in agar plates, showing the capacity of the alga to re-growth *ex-vivo* (F). RT-PCR shows the expression of the alga specific gene psbD in RNA extracts obtained from *C*. *reinhardtii* (*C*.*r*) and fishes at 3 days post injection (3 dpi). No signal was detected in the non-injected fish at 3 days post (3dpf; D). Scale bar represents 500 μm in A and 50 μm in B-D and 1.5 cm in F. Arrow heads in A indicate the areas shown in B-D. n ≥ 5 in A-D and n = 3 in F and G.

After confirming that microinjection of *C*. *reinhardtii* into the one cell stage embryo did not overtly affect development during the first few days, we decided to evaluate the distribution and viability of microalgae in injected embryos and larvae. At all of the stages examined (up to day 5 post fertilization), algae could be seen under bright field illumination as well-defined green cells. In order to visualize algae in more detail, larvae were observed by confocal microscopy, showing the presence of intact algae in a variety of host tissues (Fig 4A–4C). Further efforts to evaluate the viability of *C*. *reinhardtii*, lead us to investigate mRNA isolated from injected larvae and RT-PCR analysis was performed to detect the alga-specific transcript *psbD* (encoded by the *photosystem II reaction center polypeptide D2* gene, expressed in chloroplasts). The result of the PCR analysis showed that psbD mRNA can be detected in fish until at least 3 dpf (Fig 4D). Thus, algae seem to remain metabolically active and resided within the larva for an extended period.

**Fig 5:**
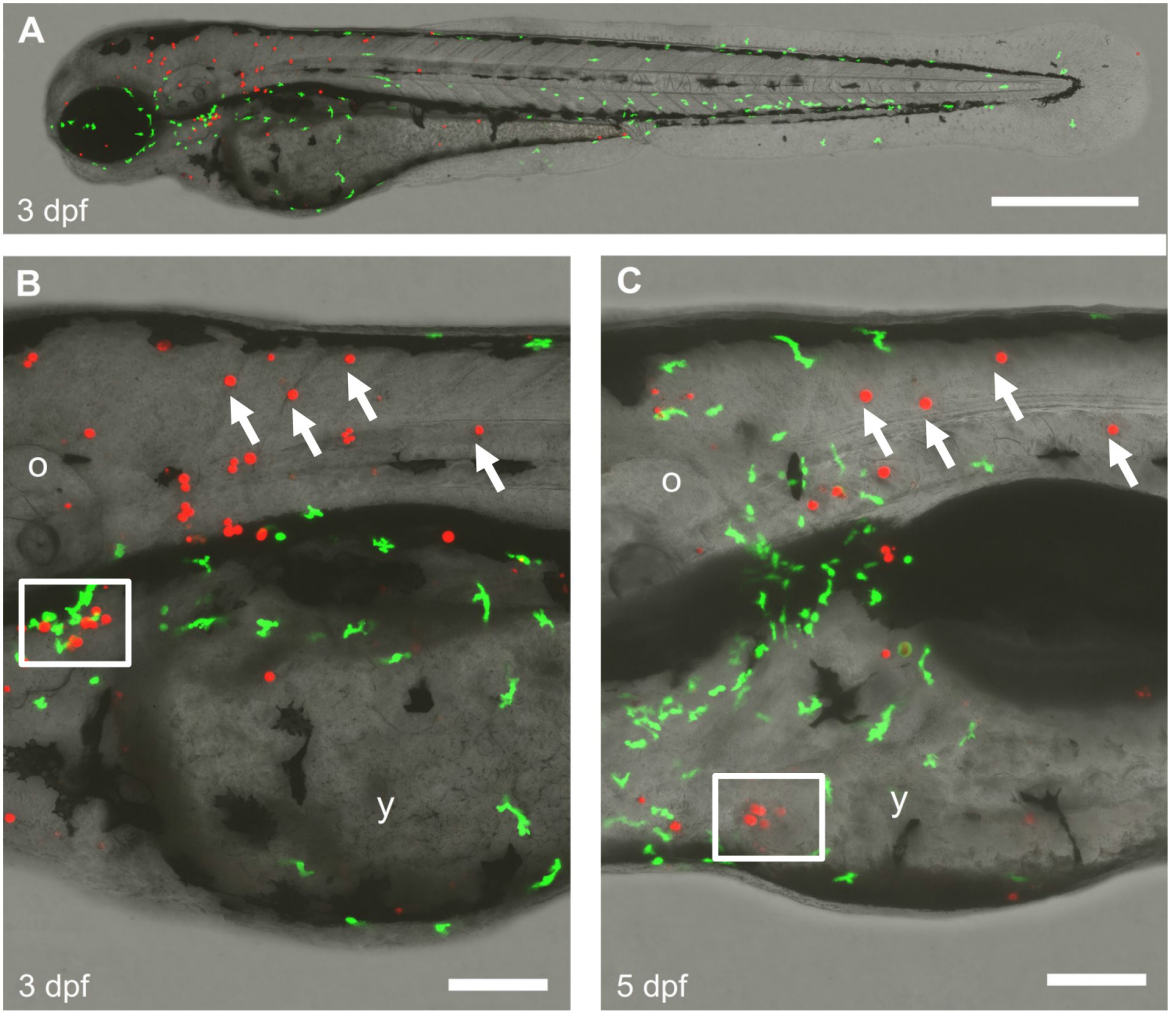
Interaction of algae with the host innate immune system. *C*. *reinhardtii* was injected in a BACmpx:GFP transgenic zebrafish embryo and raised until 3 days post fertilization (dpf). Larvae were imaged by confocal microscopy using the green and red channels to visualize the neutrophils (GPF) and algae (autofluorescence) respectively (A). A close up of the same region of the trunk is shown at 3 and 5 dpf (B-C). White arrows in B and C indicate *C*. *reinhardtii* persisting cells. The lower white square in B shows a group of cells cell that are not further seen in C, while the white square in C shows the opposite. The scale bar represents 500 μm in A and 100 μm in B-C. o, otic vesicle; y, yolk sack. n ≥ 3.

As the foreign *C*. *reinhardtii* appeared to be distributed in many different tissues, we asked whether the fish immune system interacts with microalgae. We injected a suspension of algae into BAC*mpo*::*GFP* transgenic zebrafish embryos. In these fish, innate immune leukocytes are labeled with GFP [5], which allows observation of inflammatory responses or interaction of the leukocytes with infiltrating microorganisms. In this experiment, we visualized algae by virtue of their red autofluorescence, allowing us to monitor the interaction with host leukocytes, labeled with GFP. The examination of injected fish at 48 and 96 hpf did not revealed any significant inflammatory response, though some leukocytes were found in the vicinity of *C*. *reinhardtii* cells (Fig 5). In some instances, we observed leukocytes located in the vicinity of cell debris that contained red autofluorescence, possibly indicating that immune cells may generate a cytotoxic response in the presence of *C*. *reinhardtii*. However, many microalgae still appear intact at 5 days post injection, proving that *C*. *reinhardtii* cells can escape the host’s immune response for at least this length of time.

## Discussion

The extreme dependency to an external oxygen supply observed in animals, represents a serious clinical problem as several pathological conditions are related to temporary low oxygen tension in tissues. Ischemia reperfusion injuries, chronic wounds and fibrosis are among the most common ones [11–14]. In this work we wanted to incorporate photosynthetic eukaryotic cells into a vertebrate animal model, thus contributing to the establishment of hybrid plant-animal systems [15–17]. In this work, we injected the microalga *C*. *reinhardtii* into zebrafish eggs and observed survival of both the plant cells and the animal host. *C*. *reinhardtii* is a single cell microalga of about 10 μm in diameter, used as a model organism in different fields of research including circadian rhythms [18], ciliary motility [19], photosynthesis [20] and bio fuel production [21]. On the other hand, zebrafish represent one of the best-studied organisms for developmental biology research. Due to its rapid development, relatively easy manipulation and optical transparency in early stages it also represents an ideal model for engineering photosynthetic vertebrates.

A significant rate of mortality was observed with increased amounts of algae (Fig 1A and 1B). Toxicity of the alga medium could be discarded as the control group contains only TAPS. Such mortality may partially occur due to a physical interference of the algae with early fish development. This hypothesis is supported by the increased survival observed when the algae were injected into the yolk cell at 24 hpf. This time is far beyond the mid-blastula transition, and when there is no longer active yolk cell to embryo transport, and thus the injected cells remain mainly accumulated in the yolk sphere. Although algae did not migrate into the embryo, high concentrations of algae caused higher mortality rates, suggesting that the presence of large numbers of *C*. *reinhardtii* cells in the yolk may cause the accumulation of metabolites, oxidative stress toxicity, or other factors that may affect embryonic development. An intriguing possibility is that large amounts of algae decrease hypoxia, which has been described as a key process for embryonic development.

Injected microalgae follow the ooplasmic movements and transport pathways utilized by endogenous material in early stages of zebrafish development [9, 10]. Interestingly, *C*. *reinhardtii* appears not to be recognized as a foreign body by the embryo, thus crossing the yolk cell/ blastodisc boundary region, and becoming incorporated within the cells of the blastodisc. While we occasionally saw clumps of algal cells, they were usually randomly distributed as single cells throughout the embryo and larval body, they seemed to localize subepidermally and did not seem to move over time, except for occasions in which they entered the blood flow. While we observed apparently intact *C*. *reinhardtii* cells within the fish larvae, we sought an additional means to confirm that they were still viable. We reasoned that the presence of *psbD* mRNA transcripts in the sample may be considered indicative of metabolic activity (and thus survival) of microalgae in the fish body (Fig 4B). This conclusion is further supported by the observation that injected microalgae can re-gowth *ex-vivo* from extracts obtain 3 days post injection (Fig 4C). Interestingly, several algae were observed in pairs, suggesting the possibility of replication within the fish body (Fig 5B). Other cells from a variety of sources have been successfully injected into zebrafish embryos. Besides xenografts and transplantation of mammalian cells to study tumor formation [22], a recent report describes the injection of photosynthetic cyanobacteria into zebrafish eggs [16]. Additionally, others have used this model to study bacterial infectious diseases and immune function by injecting a wide diversity of bacteria and viruses [23].

It has been reported that *C*. *reinhardtii* does not harbor any known pathogenic virus or other harmful molecules [8] and hence is generally recognized as safe (GRAS) by the Food and Drug Administration. This may partially explain why microalgae were well tolerated by the embryo. Interestingly, intact algae were continuously observed in the fish, showing that the immune response may not represent an insurmountable obstacle to generate photosynthetic vertebrates (Fig 5). Besides the immune tolerance exhibited by the host, the chimerical vertebrate described in this paper is also possible because algae provide the appropriate microenvironment to the chloroplasts. Such phenomenon differs from that present in other photosynthetic associations where the chloroplasts are taken from the plant cells, to be incorporated in the animal cells as “kleptoplastids” [24]. Those associations are far more complex because most of the chloroplast proteins are encoded in the nucleus rather than in the plastid itself.

A thrilling possibility is that algae could be engineered to produce metabolites other than oxygen and transfer them to the host. For instance, *C*. *reinhardtii* strains expressing recombinant proteins could be incorporated into the fish, where they could provide growth factors or other molecules of biotechnological interest. An advantage of this scheme is that algae would remain viable while the fish are exposed to light and/or remain transparent. At later stages, even if algae should remain in the interior of the animal, a period of darkness could ensure their disappearance.

In this work, we present evidence supporting the idea that chimerical plant-vertebrate organisms (plantebrates) can be engineered by injecting microalgae into early stage zebrafish embryos. However, further studies should be performed to provide more functional and long term data to evaluate whether the incorporation of algae into vertebrates increases their independence of an external oxygen supply and whether the symbiotic algae might also contribute to the host by generating chemical energy from their photosynthetic activity.

## Supporting Information

S1 Movie Migration of the algae to the embryo. *C*. *reinhardtii* (white and round structures) was injected in the yolk sack and quickly moves to the animal pole, being incorporated in the cellular mass of the embryo.
(AVI)
